# Signal Factors Secreted by 2D and Spheroid Mesenchymal Stem Cells and by Cocultures of Mesenchymal Stem Cells Derived Microvesicles and Retinal Photoreceptor Neurons

**DOI:** 10.1155/2017/2730472

**Published:** 2017-01-18

**Authors:** Lili Xie, Mao Mao, Liang Zhou, Lusi Zhang, Bing Jiang

**Affiliations:** ^1^Department of Ophthalmology, The Second Xiangya Hospital, Central South University, Hunan Province, China; ^2^Departments of Ophthalmology and Anatomy, Institute for Human Genetics, UCSF School of Medicine, San Francisco, CA, USA

## Abstract

We aim to identify levels of signal factors secreted by MSCs cultured in 2D monolayers (2D-MSCs), spheroids (spheroids MSCs), and cocultures of microvesicles (MVs) derived from 2D-MSCs or spheroid MSCs and retinal photoreceptor neurons. We seeded 2D-MSCs, spheroid MSCs, and cells derived from spheroids MSCs at equal numbers. MVs isolated from all 3 culture conditions were incubated with 661W cells. Levels of 51 signal factors in conditioned medium from those cultured conditions were quantified with bead-based assay. We found that IL-8, IL-6, and GRO*α* were the top three most abundant signal factors. Moreover, compared to 2D-MSCs, levels of 11 cytokines and IL-2R*α* were significantly increased in conditioned medium from spheroid MSCs. Finally, to test if enhanced expression of these factors reflects altered immunomodulating activities, we assessed the effect of 2D-MSC-MVs and 3D-MSC-MVs on CD14+ cell chemoattraction. Compared to 2D-MSC-MVs, 3D-MSC-MVs significantly decreased the chemotactic index of CD14+ cells. Our results suggest that spheroid culture conditions improve the ability of MSCs to selectively secrete signal factors. Moreover, 3D-MSC-MVs also possessed an enhanced capability to promote signal factors secretion compared to 2D-MSC-MVs and may possess enhanced immunomodulating activities and might be a better regenerative therapy for retinal degenerative diseases.

## 1. Introduction 

There has been considerable interest in the curative effect of the human mesenchymal stem cells (MSCs) that are derived from adult tissues such as umbilical cord blood, bone marrow, and adipose tissue [1–3]. MSCs are relatively convenient to be isolated from donors, and they can maintain an active proliferating capacity after multiple passages in culture. For these reasons, MSCs have great therapeutic potential in disease treatment, as demonstrated by results from multiple experimental and clinical studies [4–6]. In addition to their multidifferentiation potential, MSCs are well known for their abilities to secrete paracrine factors and to modulate inflammation and immunity [7, 8]. Interestingly, therapeutic effects are frequently reached without convincing evidence of cell differentiation or engraftment in vivo [9]. Instead, MSCs contribute to tissue repair through secretion of a certain set of paracrine factors with limited tissue destruction, thus showing broad application prospect in treatment of multiple diseases including those involving retinal degeneration [10, 11]. Previously investigated neuroprotective therapies for degenerative retinal photoreceptor neurons such as age-related macular degeneration (AMD) and retinitis pigmentosa (RP) include intravitreal MSC transplantation, MSC conditioned medium injection, and neurotrophic factor delivery [12, 13]. However, the exact therapeutic mechanism and factors determining the curative effect remain to be fully elucidated. Increasing attention has been paid to ways to enhance MSC treatment efficiency and to identify MSC-derived elements conferring potent neuroprotection [14].

Traditionally, MSCs were cultured in 2D monolayers (2D-MSCs) [15]. Recently, aggregation of MSCs into 3D spheroids (spheroid MSCs) was reported to show increased therapeutic potentials than 2D-MSCs, in part, because they better mimic the structure and arrangement of a real tissue [16, 17]. Similarly, microvesicles (MVs) derived from MSCs (MSC-MVs), which function as shuttles for cytokines, receptors, ligands, message RNAs (mRNAs), microRNAs (miRNAs), and lipids [18, 19], were shown to be mediators of cell-to-cell communication [20]. MSC-MVs were thought to be an effective therapeutic approach not merely because they are stable and preservable, but also because they have less potential risks of immunological rejection [21, 22]. However, most studies so far have only utilized MVs derived from 2D-MSCs (2D-MSC-MVs), and advantages of using MVs derived from spheroid MSCs (3D-MSC-MVs) in disease treatment have not been fully explored.

Our study aimed to systematically analyze signal factors secreted by 2D-MSCs and spheroid MSCs and the effect of 2D-MSC-MVs and 3D-MSC-MVs on signal factor secretion when cocultured with retinal photoreceptor neurons. Our results suggested that the 3D culture model enhanced the ability of MSCs to secrete signal factors responsible for anti-inflammation, cell differentiation, and cell survival, and 3D-MSC-MVs possessed enhanced capability of promoting signal factors secretion and may possess enhanced immunomodulating activities compared to 2D-MSC-MVs and might be a better option for neuroprotection of retinal photoreceptor neurons.

## 2. Materials and Methods

### 2.1. MSC Cell Culture

Passage 2 MSCs derived from human umbilical cord blood were obtained from Cyagen Biosciences Inc. (Guangzhou, China). MSCs were cultured in Dulbecco's modified Eagle Medium (DMEM) (Cyagen) supplemented with 10% fetal bovine serum (FBS) (Cyagen), 1% penicillin/streptomycin, and 1% L-glutamine. Cells were cultured at 37°C with 5% CO_2_ in a humidified incubator.

### 2.2. Spheroid Generation and Dissociation

**Figure 1:**
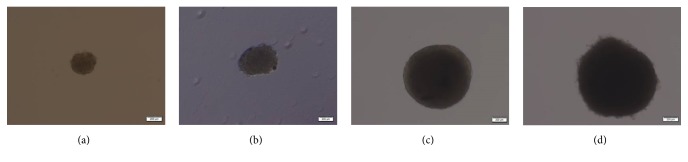
Gathering of MSCs into spheroids. Microscopic images showing MSCs seeded at four different concentrations (2.5 × 10^3^, 6.25 × 10^3^, 25 × 10^3^, and 50 × 10^3^ cells/drop) aggregated into spheroids different in size in a hanging drop. Scale bar = 200 um.

**Figure 2:**
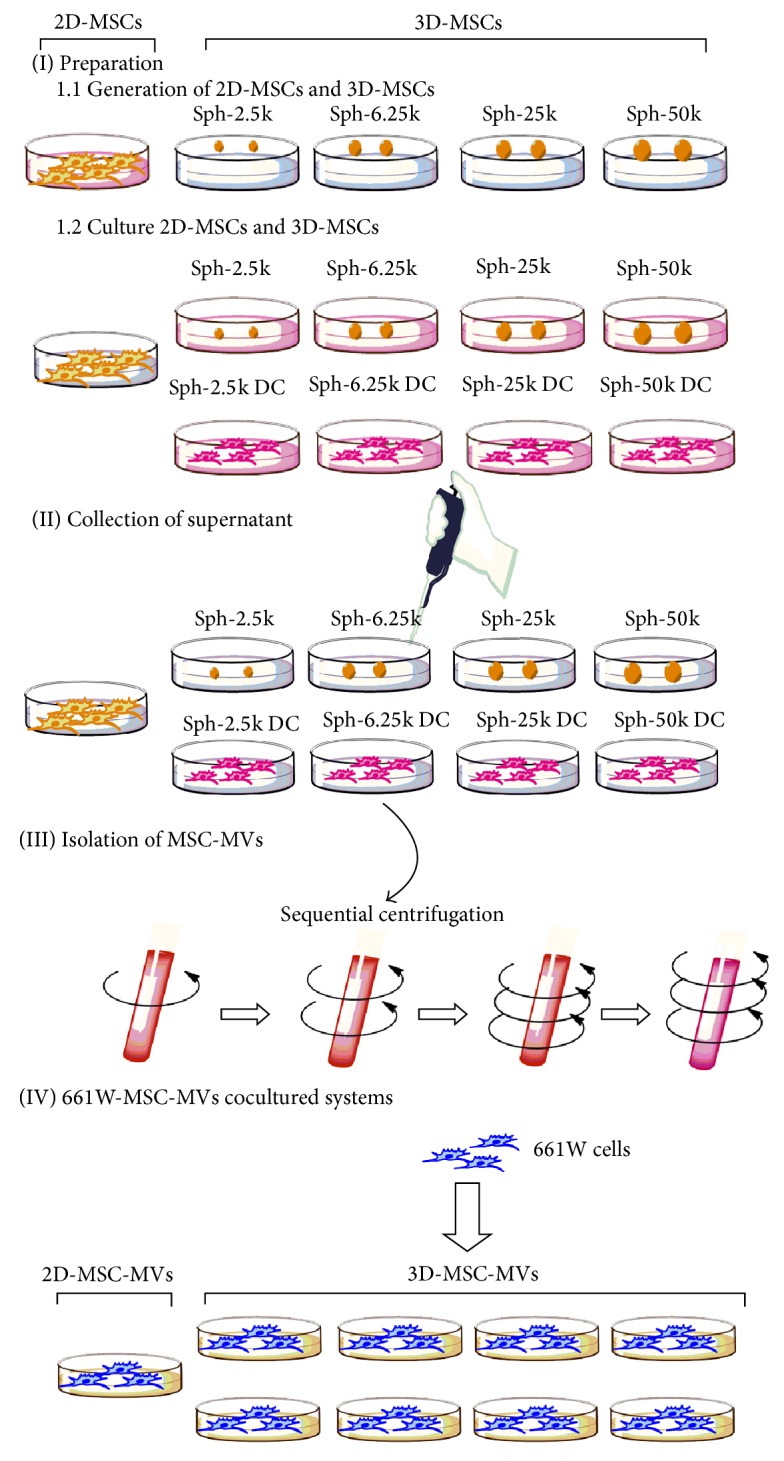
Schematic illustration of the experimental design. A part of MSCs were cultured in 2D environment (2D-MSCs) and hanging drop protocol was used to prepare spheroid MSCs (I). Different forms of conditioned medium were collected after 24 h (II). MSC-MVs were isolated from conditioned medium of MSCs based on sequential centrifugation (III). 2D-MSC-MVs/3D-MSC-MVs were cocultured with 661W cells for different time period (IV).

A hanging drop protocol was used for generation of spheroid MSCs as described by Bartosh et al. [16]. Briefly, MSCs were plated on an inverted culture dish lid as hanging drops in 35 ul of conditioned culture medium at four different cell densities (2.5 × 10^3^, 6.25 × 10^3^, 25 × 10^3^, and 50 × 10^3^ cells/drop and hereafter referred to as Sph-2.5k, Sph-6.25k, Sph-25k, and Sph-50k, resp.) (Figure 1). The lid was then flipped and placed onto a culture dish into which PBS were injected to prevent evaporation. Hanging drop cultures were grown at 37°C with 5% CO_2_ for 72 h to generate spheroids. MSC spheroids were harvested with a cell lifter and transferred to a 15 mL centrifuge tube with PBS and collected by centrifugation at 1,000 rpm for 5 min. In order to obtain spheroid derived MSCs, spheroids were incubated with 0.25% trypsin and 1 mM EDTA (Gibco-Invitrogen, California, USA) at 37°C for different time periods depending on the spheroid size (1, 2, 10, and 15 min for Sph-2.5k, Sph-6.25k, Sph-25k, and Sph-50k, resp.). Spheroid derived MSCs were collected by centrifugation at 1,000 rpm for 5 min before being used in follow-up assays. Hereafter, spheroid derived MSCs were referred to as Sph-2.5k DC, Sph-6.25k DC, Sph-25k DC, and Sph-50k DC, respectively, depending on the size of original spheroids (Figure 2).

### 2.3. Collection of Conditioned Medium

Equal total numbers of 2D-MSCs, spheroid MSCs, and spheroid derived MSCs (50 × 10^3^ cells/well) were seeded onto six-well dishes in 2 mL serum-free medium. Conditioned medium from different forms of MSCs was collected after 24 h in culture.

### 2.4. Isolation of MSC-MVs

MSC-MVs were isolated from serum-free conditioned medium from different forms of MSC cultures (Figure 2) following a standard sequential centrifugation protocol [23]. Briefly, conditioned medium was centrifuged at 2,000 ×g for 20 min at 4°C to get rid of cells and cell debris. The supernatants were then centrifuged at 100,000 ×g (Beckman Coulter Optima L-100 XP Ultracentrifuge, Beckman Coulter, California, USA) for 1 h at 4°C, and the pellets were washed once in PBS. The resulting supernatants were ultracentrifuged again at 100,000 ×g for 1 h at 4°C to collect MSC-MVs. The MSC-MVs collected from a total of 1 × 10^6^ cells were suspended in 100 *μ*L PBS and then suspended in 1 mL serum-free medium.

### 2.5. Electron Microscopy

MSC-MVs purified by ultracentrifugation were applied to carbon-coated grids and stained with 1% uranyl acetate. The grids were examined by Tecnai G2 Spirit TWIN Transmission electron microscope (FEI) at an acceleration voltage of 80 kV. Photographs were taken with an AMT 2k CCD camera.

### 2.6. Total Protein Quantification

Concentrations of total proteins from MSC-MVs suspended in 100 *μ*L PBS were detected using Pierce TM BCA Protein Assay Kit (23225) (Thermo Scientific™, Rockford, USA) according to the manufacturer's protocol. Data were acquired using Epoch™ Multi-Volume Spectrophotometer system (BioTek, California, USA).

### 2.7. Coculture of 661W Cells and MSC-MVs

The 661W cell line was a gift from the Zhongshan Ophthalmic Center, Zhongshan University. For the 661W-MV coculture experiment, 1 × 10^5^ 661W cells were treated with 2 mL of 2D-MSC-MVs, 2 mL of Sph-2.5k-MVs, 2 mL of Sph-2.5k DC-MVs, 2 mL of Sph-6.25k-MVs, 2 mL of Sph-6.25k DC-MVs, 2 mL of Sph-25k-MVs, 2 mL of Sph-25k DC-MVs, 2 mL of Sph-50k-MVs, and 2 mL of Sph-50k DC-MVs, respectively (Figure 2), and were cultured at 37°C with 5% CO_2_ for different time points (24 h, 48 h, 72 h, and 96 h) in 6-well plates. 661W cells treated with 2 mL serum-free medium without MSC-MVs were used as controls. The conditioned medium from each culture condition was then collected and assayed for signal factors.

### 2.8. Bead-Based Analysis

Quantification of signal factors was performed using the Bio-Plex Pro™ TGF*β* Assays Kit (171W4001 M), Bio-Plex Pro™ Human Cytokine 27-Plex Assays Kit (M500KCAF0Y), and Bio-Plex Pro™ Human Cytokine 21-Plex Assays Kits (MF0005KMII) (Bio-Rad, California, USA) according to manufacturer's instructions. Conditioned medium collected from different culture and coculture conditions described above were analyzed for signal factors listed in Table S1 (see Supplementary Material available online at https://doi.org/10.1155/2017/2730472). Each sample was run in triplicate. Data were acquired using the Bio-Plex 200 system (Bio-Rad, California, USA) and standard curves were generated by 5-parametric curve fitting using the OriginPro 8.5.0 software (OriginLab, Massachusetts, USA).

### 2.9. CD14+ Cell Isolation

Fresh peripheral blood samples were collected from 5 health care workers. All subjects provided written informed consent and the study was approved by the human ethics committee of the Second Xiangya Hospital of Central South University, prior to the initiation of study. Peripheral blood mononuclear cells (PBMC) were isolated from heparinized venous peripheral blood by using Ficoll-Hypaque density gradient centrifugation (GE Health care, Switzerland) for 30 min at 500 g. CD14+ cells were selected from PBMC by positive selection using anti-CD14 conjugated microbeads (Miltenyi Biotec, San Diego, CA, USA) under endotoxin-free conditions, according to the manufacturer's instructions. The purity of these isolated cell populations was tested by flow cytometry. Isolated cells were incubated with the fluorescent-labeled antibodies against CD14 (BioLegend, San Diego, USA) for 30 min on 4°C in dark. Flow acquisition was performed on FACSCantoTM II analyzer (BD, CA, USA). The data were analyzed using FlowJo 7.6 software (Tree Star Inc., CA, USA) and the purity of these isolated cell populations was higher than 90% (Figure 6(f)).

### 2.10. Migration Assay

The migration assay for CD14+ cells was performed using 8 *μ*m pore size polycarbonate membrane (Corning Inc., CA, USA). Two hundred microliters of CD14+ cells in serum-free RPMI-1640 medium (Gibco, Carlsbad, CA, USA) (5 × 10^4^ cells) was placed into the upper insert and 4 different kinds of medium were added to the lower chambers, respectively: 600 *μ*L of serum-free RPMI-1640 medium, 500 *μ*L of serum-free RPMI-1640 medium supplemented with 100 *μ*L of poly(I:C) solution (1 mg/mL in PBS; Enzo, Farmingdale, NY, USA), 400 *μ*L of serum-free RPMI-1640 medium supplemented with 100 *μ*L of poly(I:C) solution and Sph-25k-MVs collected from 1 × 10^6^ cells suspended in 100 *μ*L PBS, 400 *μ*L of serum-free RPMI-1640 medium supplemented with 100 *μ*L of poly(I:C) solution, and 2D-MSC-MVs collected from 1 × 10^6^ cells suspended in 100 *μ*L PBS. After culturing in a humidified CO_2_ incubator at 37°C for 2 h, cells on the top were removed and the CD14+ cells migrating across the membrane were fixed with methanol, stained with 0.5% crystal violet. Cells were quantitated in 6 random fields and averages from these fields were calculated. Experiments were repeated three times and results were presented as chemotactic index (CI), defined as the number of migrated cells divided by number of migrated cells responding to the serum-free RPMI-1640 medium. The optical density of each well was determined using Centrifugal Ultra Filters (Millipore, Billerica, USA) set to 450 nm.

### 2.11. ELISA Assay

Conditioned media from different forms of MSCs and 661W-MSC-MVs coculture system were analyzed for VEGF and TGF*β*3 using Human VEGF Quantikine ELISA Kit (DVE00) and human TGF*β*3 DuoSet ELISA Kit (DY243) (R&D System, Minneapolis, USA) according to manufacturer's instructions, respectively. Samples were run in triplicate.

### 2.12. Statistical Analyses

Heatmaps were generated through taking an average of each group (*n* = 3) and plotted in line with a predetermined color code using HemI 1.0 [24]. One-way ANOVA was used to analyze differences among samples from 2D and 3D groups, and then post hoc tests were performed to compare difference between two groups. To analyze different expression levels among samples from various time points, independent-samples *T* test was applied and the *p* value less than 0.05 was considered as statistically significant. Data are expressed as mean ± standard deviation of the mean, unless otherwise stated. These analyses were performed using Prism software 5.0 (GraphPad Software, La Jolla, USA).

## 3. Results

### 3.1. IL-8, IL-6, and GRO*α* Are the Top Three Cytokines in Concentration Secreted by 2D-MSCs

MSCs are known to have paracrine effects by secretion of signal factors such as interleukins, chemokines, and growth factors. To analyze signal factors secreted by 2D-MSCs, conditioned media of 2D-MSCs were collected and 51 signal factors (Table S1) in conditioned medium were quantified by a multiplex bead-based assay. These signal factors could play a part in regulation of inflammation, differentiation, and growth, and they are likely to affect the development and treatment of degenerative disease [25, 26]. In the 2D culture condition, a total of 15 signal factors secreted by MSCs were detectable (Table 1) and IL-8, IL-6, and GRO*α* are the top three signal factors in concentration. Furthermore, we found TGF*β*1, bFGF, SCGF-*β*, and HGF are highly expressed, which are cytokines involved in regeneration, proliferation, and differentiation. In contrast, proinflammatory factors such as MIP-1*α*, MIP-1*β*, TNF-*α*, TNF-b, and IFN-*γ* and certain chemokines such as RANTES, MIG, and IP-10 were not detected. Interestingly, in addition to a series of cytokines, conditioned media of 2D-MSCs also contain a cytokine receptor, IL-2R*α*, which is an integral IL-2 receptor.

### 3.2. Spheroid MSCs Showed Enhanced Secretion of 11 Cytokines and 1 Cytokine Receptor Compared to 2D-MSCs

Spheroid MSCs have been shown to have improved therapeutic effects compared to monolayer cultures because of their enhanced anti-inflammatory properties, differentiation capacity, and cell survival [16, 27, 28]. To understand the potential mechanism, we also investigated secretion of signal factors from spheroid MSCs. Since culture conditions such as spheroid size can influence MSC behaviors [29], we prepared spheroid MSC cultures with different sizes (Sph-2.5k, Sph-6.25k, Sph-25k, and Sph-50k groups for cells seeded at 2.5 × 10^3^, 6.25 × 10^3^, 25 × 10^3^, and 50 × 10^3^ cells/drop, resp.). Moreover, it was reported that individual MSCs isolated from spheroids are smaller in size and had increased anti-inflammatory capacity compared to 2D-MSCs that never formed aggregates or spheroids, thus being an attractive therapeutic tool [16]. Therefore, we also investigated secretion of spheroid derived MSCs that were released from spheroids by trypsinization (Sph-2.5k DC, Sph-6.25k DC, Sph-25k DC, and Sph-50k DC groups for corresponding spheroid sizes of 2.5 × 10^3^, 6.25 × 10^3^, 25 × 10^3^, and 50 × 10^3^ cells/drop, resp.) in this study. Among the 51 signal factors analyzed, a total of 21 signal factors were detected with the bead-based assay including the 15 signal factors that were also detected in 2D-MSCs cultures (Table 2). Six signal factors, IL-1Ra, IL-7, IL-16, MCP-3, TGF*β*3, and VEGF, were detected only under 3D culture conditions.

**Figure 3:**
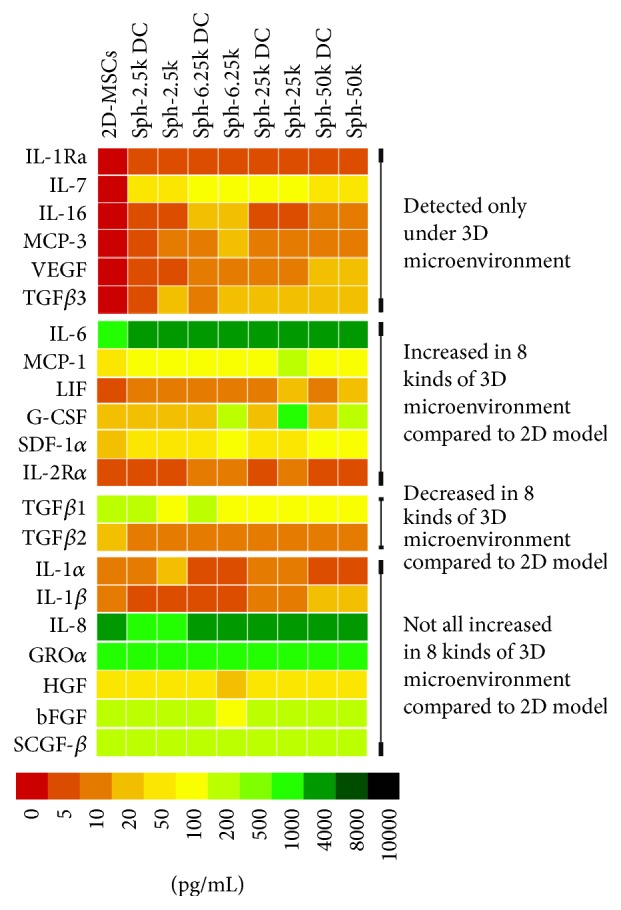
Heatmap of signal factor secretion by 2D-MSCs and 3D-MSCs cultured at different conditions. Six signal factors, IL-1Ra, IL-7, IL-16, MCP-3, TGF*β*3, and VEGF, were detected only under 3D culture conditions. 5 signal factors, IL-6, MCP-1, LIF, G-CSF, and SDF-1*α*, were higher in conditioned medium from all 3D culture conditions compared to the 2D culture condition. Two signal factors, TGF*β*1 and TGF*β*2 had decreased levels in conditioned media from 3D culture conditions compared to the 2D culture condition.

Next, we compared levels of signal factors that were both detectable in conditioned medium from spheroid MSCs and 2D-MSCs (Figure 3). We found that levels of 5 signal factors, IL-6, MCP-1, LIF, G-CSF, and SDF-1*α* were higher in conditioned medium from all 3D culture conditions compared to the 2D culture condition. Apart from increased secretion of these 11 signal factors that are involved in inflammatory regulation, cell differentiation, and survival, aggregation of MSCs into 3D spheroids enhanced secretion of a cytokine receptor, IL-2R*α*. Moreover, we found that cell densities in spheroids and culture methods affected levels of certain signal factors. Highest levels of IL-7, IL-16, MCP-3, and IL-2R*α* were detected in the Sph-6.25k group. Similarly, highest levels of MCP-1, LIF, and G-CSF were detected in the Sph-25k group, and highest levels of IL-1Ra, TGF*β*3, SDF-1*α*, and VEGF were detected in the Sph-50k group. In addition, levels of these signal factors tended to be higher in spheroid MSC groups than their corresponding spheroid derived MSCs groups, suggesting the microenvironment within the 3D spheroids promotes certain signal factor secretion. Furthermore, the expressions of VEGF and TGF*β*3 in supernatants of 2D-MSCs/3D-MSCs were also evaluated by ELISA (Figure S1. A, C). Consistent with the results from the bead-based assay, VEGF and TGF*β*3 were detected only under 3D culture conditions and their highest levels were detected in the Sph-50k group and Sph-25k group, respectively.

### 3.3. 3D-MSC-MVs Possessed Enhanced Capability of Promoting Signal Factors Secretion Compared to 2D-MSC-MVs

**Figure 4:**
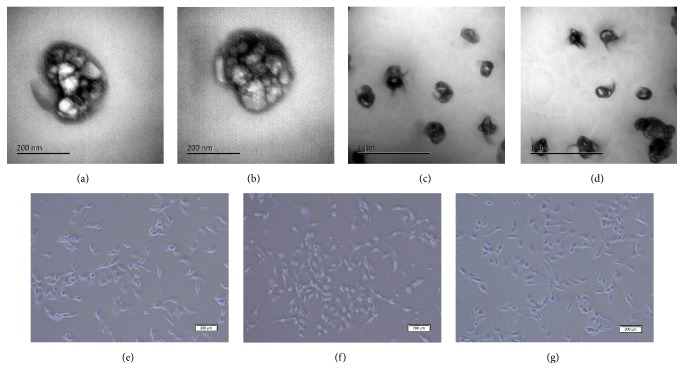
Characteristics of 2D-MSC-MVs and 3D-MSC-MVs. (a)–(d) By electron microscopy, both 2D-MSC-MVs (a, c) and 3D-MSC-MVs (b, d) displayed sizes ranging from 100 nm to 400 nm. Scale bar = 200 nm, 1 um. (e)–(g) Light microscopy analyses showed that, similar to controls (e), both of 2D-MSC-MVs (f) and 3D-MSC-MVs (g) could maintain the normal shape of cocultured 661W cells (scale bar = 200 um).

**Figure 5:**
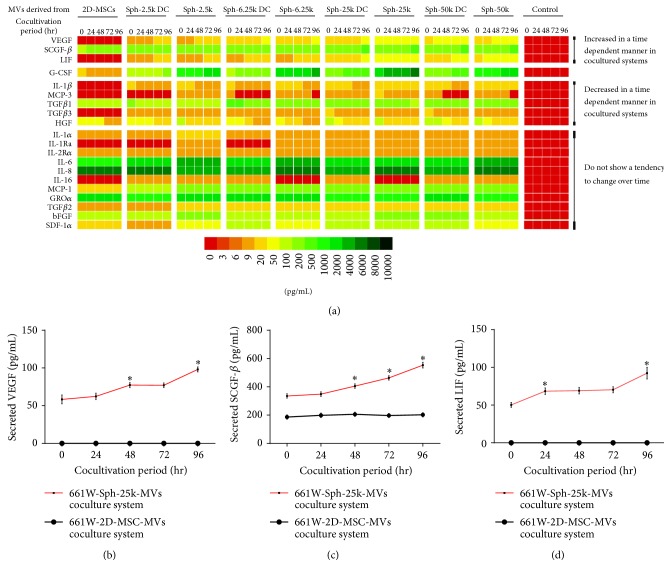
Heatmap of signal factor secretion by MVs derived from 2D-MSCs or 3D-MSCs cocultured with 661W cells. (a) 3D-MSC-MVs possessed enhanced capability of promoting signal factors secretion compared to 2D-MSC-MVs. SCGF-*β*, VEGF, and LIF expression levels are increased in a time dependent manner in supernatants from 661W-3D-MSC-MVs cocultured systems (b, c, d). *∗* denotes significant differences of *p* value less than 0.05.

**Figure 6:**
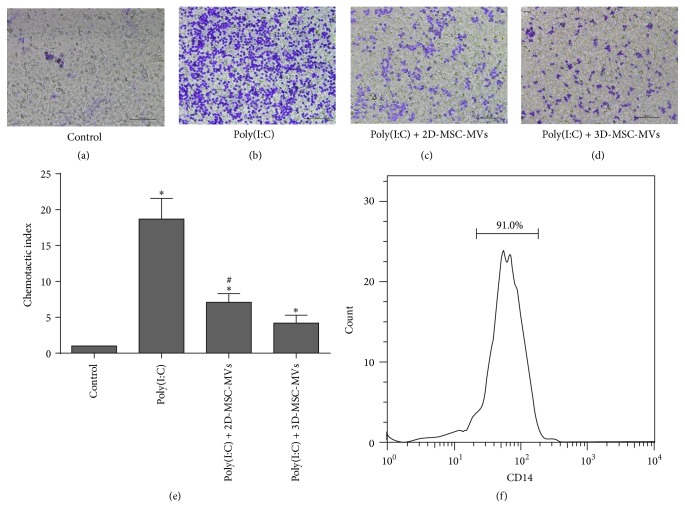
The chemotactic activity of poly(I:C) to CD14 cells and the influence of 2D-MSC-MVs and 3D-MSC-MVs on CD14 cells migration. (a)–(d) Light microscopic images showing poly(I:C) solution (b) significantly induced the CD14 cells migration and compared to 2D-MSC-MVs (c), 3D-MSC-MVs (d) could significantly decrease the migration of CD14 cells. Scale bar = 100 um. (e) Quantification of transwell migration of CD14 cells. (f) Through flow cytometry, the purity of CD14+ cells was validated and higher than 90%. *∗* denotes significant differences between control and each group of *p* value less than 0.05. # denotes significant differences between 2D-MSC-MVs and 3D-MSC-MVs group of *p* value less than 0.05.

Since MSC-MVs can serve as effective shuttles of bioactive molecules, thus mediating MSC effects and modulating activities of receipt cells, we cocultured MSC-MVs with 661W cells and assessed influence of MSC-MVs on 661W cells. To this end, we isolated MVs from conditioned medium of both 2D-MSC and 3D-MSC cultures (including spheroid MSCs and spheroid derived MSCs with 4 different cell densities, namely, Sph-2.5k, Sph-6.25k, Sph-25k, and Sph-50k and Sph-2.5k DC, Sph-6.25k DC, Sph-25k DC, and Sph-50k DC groups) containing equivalent amount of total proteins and incubated them with 661W cells for different time periods. By electron microscopic analyses, both 2D-MSC-MVs and 3D-MSC-MVs displayed sizes ranging from 100 nm to 400 nm (Figures 4(a)–4(d)). Moreover, neither 2D-MSC-MVs nor 3D-MSC-MVs affected the morphology of 661W cells (Figures 4(e)–4(g)). We next quantified levels of signal factors in the conditioned medium collected from the coculture systems at 24 h, 48 h, 72 h, and 96 h after incubation using the bead-based assay. A total of 20 signal factors were detectable under at least one of the coculture conditions (Figure 5(a)). Similar to results we observed from MSCs, MVs from 3D-MSCs tend to have a stronger effect on signal factor secretion. Among the 20 signal factors, 11 were found to have higher levels in all 661W-3D-MSC-MVs coculture conditions than 661W-2D-MSC-MVs. For the remaining 9 signal factors, their concentrations were higher in at least 1 of 8 661W-3D-MSC-MVs coculture conditions than in the 661W-2D-MSC-MVs coculture condition. Moreover, cell densities of spheroids also affected secretion profiles of signal factors. Among the 20 signal factors, 11 signal factors were found to have the highest levels in the 661W-Sph-25k-MVs group and 6 were highest in the 661W-Sph-6.5k-MVs group. Similarly, for the remaining 3 signal factors, one had highest levels in the 661W-Sph-2.5k-MVs group and 2 were in the 661W-Sph-50k-MVs group. Interestingly, levels of some of these signal factors showed a pattern of either increase or decrease along with cultivation time. Three signal factors, SCGF-*β*, VEGF, and LIF showed an increase with cocultivation time in 661W-3D-MSC-MVs coculture systems (Figures 5(b)–5(d)). On the other hand, different behavior was observed for IL-1*β*, MCP-3, TGF*β*1, TGF*β*3, and HGF in which a time dependent decrease was detected in all 9 coculture systems. Levels of other 11 cytokines in the conditioned medium from cocultured 661W-MSC-MVs did not have an obvious trend to change over time. Interestingly, along with the increase of cocultivation time, G-CSF levels in 661W-2D-MSC-MVs group were sustainably decreased; however, its levels were gradually increased with time in all 661W-3D-MSC-MVs cocultured groups, supporting that different sources of MSC-MVs could affect the interaction between MSC-MVs and 661W cells. Moreover, for 661W-3D-MSC-MVs coculture condition, MVs from Sph-2.5k, Sph-6.25k, Sph-25k, and Sph-50k tend to have a stronger effect on SCGF-*β*, VEGF, LIF, and G-CSF secretion than MVs from Sph-2.5k DC, Sph-6.25k DC, Sph-25k DC, and Sph-50k DC groups correspondingly, suggesting microenvironment within the 3D spheroids promotes certain signal factor secretion. The expression of VEGF and TGF*β*3 in 661W-Sph-25k-MVs coculture system was also tested by ELISA (Figure S1. B, D). Similar to the results from bead-based assay, TGF*β*3 levels in the 661W-Sph-25k-MVs group were sustainably decreased, but VEGF levels were gradually increased with time in 661W-Sph-25k-MVs group. Finally, to evaluate if these signal factors induced by MVs may possess immunomodulation activities, we tested their effect on CD14+ cell chemoattraction using a transwell cell assay (Figure 6). CD14+ cells can respond to proinflammatory substances such as dsDNA [30]. Therefore, we first induced CD14+ cells chemoattraction by adding a dsDNA analog, poly(I:C), in the lower chamber of transwells. As expected, poly (I:C) treatment significantly increased the number of CD14+ cells migrated through the transwell membrane. We next tested the effect of MSC-MVs on CD14+ migration by adding MSC-MVs along with poly(I:C) in the lower chamber. We found that both 2D-MSC-MVs and 3D-MSC-MVs inhibited CD14+ cell migration and more importantly, 3D-MSC-MVs had a stronger antimigration effect than 2D-MSC-MVs.

## 4. Discussion

Stem cell therapy has been used to treat retinal diseases by optimizing immune responses and improving the local microenvironment [31, 32]. In addition to known growth-promoting and neurotrophic effects, MSCs have great potentials in modulation of inflammation, immunity, and regeneration through paracrine and juxtacrine actions [28, 33]. In addition, conditioned media from MSC cultures have been employed as feasible and effective materials to attenuate injury and promote retinal neuron survival both in vitro and in vivo suggesting MSC-MVs can be a potent treatment approach [34–36]. It was reported that aggregation of MSCs into spheroids could enhance their anti-inflammatory abilities by increasing secretion of anti-inflammatory factors [16]. However, detailed analyses comparing signal factor profiles between 2D-MSCs and spheroid MSCs have yet to be reported. In this study we systematically profiled and compared signal factors secreted by 2D-MSCs and spheroid MSCs. In addition, we analyzed the effect of MVs derived from either 2D or spheroid MSC cultures on signal factor secretion when cocultured with 661W cells. Our results revealed that spheroid culture models improved the ability of MSCs to secret signal factors responsible for anti-inflammation, cell differentiation, and cell survival. In addition, MVs derived from MSCs can stimulate signal factor secretion and 3D-MSC-MVs were superior to 2D-MSC-MVs in stimulating signal factor secretion.

For both 2D-MSCs and spheroid MSCs, the top three signal factors in concentration, IL-8, IL-6, and GRO*α*, play an essential role in the modulation of inflammation [37]. In addition, IL-8 is a potent angiogenic factor [38] and GRO*α* is related to the occurrence and development of certain tumors [39]. These three signal factors represent important components of MSC secretions and underline the immune modulating properties of MSCs. Moreover, spheroid MSCs are superior to 2D-MSCs as they have enhanced anti-inflammatory properties [40], enhanced differentiation capacity [41], and improved cell survival [27]. Interestingly, these enhanced capabilities of spheroid MSCs correlate with increased secretion of signal factors in the spheroid MSCs [16, 42]. In line with this notion, we have detected signal factors that were only secreted by spheroid MSCs, including IL-1Ra, IL-7, IL-16, MCP-3, TGF*β*3, and VEGF. As previously reported, these factors are important regulators of inflammation and immunity. Through inhibiting proinflammatory factors IL-1*α* and IL-1*β*, increased IL-1Ra levels can play an important role in anti-inflammation [43]. IL-7 is a growth factor for T cells and has potent proangiogenic effect [44]. IL-16 functions as a modulator of T cell activation and can inhibit HIV replication [45] and it was shown that IL-16 could prevent or delay virus infection of hypothalamus and inhibit virus from spreading into optic nerve and retina [46]. MCP-3 can attract macrophages during inflammation and metastasis and augments monocyte antitumor activity [47]. VEGF are known to promote vasculogenesis and angiogenesis and can also contribute to inflammation by promoting lymphangiogenesis [48, 49]. TGF*β*3 are involved in cell differentiation and cell survival [50] and it was shown that the hypoxic environment inside MSC spheroids promoted TGF*β*3 expression, leading to enhanced chondrogenic differentiation of MSCs and cartilage formation [51]. It is worth mentioning that, unlike TGF*β*3, two other members of the transforming growth factor beta superfamily, TGF*β*1 and TGF*β*2, had decreased secretion in conditioned medium from 3D culture conditions compared to the 2D culture condition. Thus, our results suggest that, instead of enhancing secretion of all signal factors, 3D culture model can selectively increase secretion of certain factors. Finally, our results demonstrated that MSCs properties depended largely on culture conditions. Levels of these increased signal factors tended to be higher in spheroid MSC groups than their corresponding spheroid derived MSCs groups, suggesting the microenvironment within the 3D spheroids can promote signal factor secretion. Moreover, both spheroid MSCs and spheroid derived MSCs had increased secretion of signal factors compared to 2D-MSCs, suggesting they are probably more efficient in disease treatment. In sum, our results support the notion that the selectively increased secretion of signal factors might be one of the reasons that spheroid MSCs are more advantageous than 2D-MSCs.

As mediators of bidirectional communication between MSCs and target cells, MSC-MVs have been shown to contribute greatly to tissue repair and regeneration [52–54]. In addition, signal factors play a vital role in MSC-MVs mediated cell/tissue regenerative process. For example, through RNA transfer into impaired tubular epithelial cells, MSC-MVs can stimulate hepatocyte growth factor (HGF) secretion and facilitate cell growth and regeneration [55]. Similarly, in our 66W-MSC-MVs coculture system, secretion of three signal factors, SCGF-*β*, VEGF, and LIF increased with cocultivation time. SCGF-*β*, LIF, and VEGF are important signal factors in repair and regeneration of certain tissues. SCGF-*β*, a cytokine of the C-type lectin family, was originally found as a growth factor stimulating primitive hematopoietic progenitor cell proliferation [56]. Subsequent studies confirmed that SCGF-*β* can promote the development of myeloid and erythroid colonies and interact with additional soluble mediators, such as VEGF and SCF to promote the healing process [57, 58]. LIF is primarily recognized for its ability to maintain the developmental potential of embryonic stem cells [59]. Recently it was also reported that LIF, through upregulating superoxide dismutase-3, could protect neurons from ischemic damage [60]. Other than its role in angiogenesis and anti-inflammation, secretion of VEGF by adult hippocampal neural stem and progenitor cells was recently shown to be required for maintenance of a neurogenic niche [61]. Therefore, although further work is needed to verify the effectiveness of SCGF-*β*, VEGF, and LIF in treatment of retinal diseases, sustainably increased secretion of these factors in our coculture systems suggests them to be potential targets for regenerative therapies for retinal photoreceptor neurons.

The characteristics of MVs can be affected by their cellular origin [62, 63]. For example, MVs derived from MSCs and liver resident stem cells (HLSCs) could selectively shuttle different pattern of miRNAs, thus having distinct biofunctionality [64]. As our results showed, MVs from Sph-2.5k, Sph-6.25k, Sph-25k, and Sph-50k tend to have a stronger effect on SCGF-*β*, VEGF, LIF, and G-CSF secretion than MVs from Sph-2.5k DC, Sph-6.25k DC, Sph-25k DC, and Sph-50k DC groups correspondingly, suggesting microenvironment within the 3D spheroids promotes certain signal factor secretion. We also found that those 3D-MSC-MVs possessed enhanced capability of promoting signal factors secretion compared to 2D-MSC-MVs, consistent with the difference in signal factor secretion between their cellular origins.

Chronic inflammation underlies various degenerative retinal diseases affecting photoreceptors [65]. For example, AMD is characterized by extracellular deposits (called drusen) that contain several proinflammatory substances such as double-stranded RNAs (dsRNAs) and lipoproteins [66]. dsRNAs serve as the ligand of toll-like receptor 3 (TLR3) that mediates inflammation and innate immune response [67]. Previous studies have shown that MSC-MVs could systematically ameliorate retinal injury through partially inhibiting the expression of monocyte chemoattractant protein that plays a vital role in monocyte mobilization and migration under chronic inflammation [68, 69]. Similarly, we found that MSC-MVs could inhibit CD14+ cell migration and compared to 2D-MSC-MVs, 3D-MSC-MVs had a stronger antimigration effect. The antimigratory effect might be due to the increased secretion of anti-inflammatory factors and thus might be more likely to reduce the chronic inflammation that underlies various degenerative retinal photoreceptor neurons. Therefore, our results suggest that a better regenerative therapy for retinal diseases can be achieved by using 3D-MSC-MVs instead of 2D-MSC-MVs.

In addition to cytokines, our results showed that aggregation of MSCs into spheroids could enhance the expression level of a cytokine receptor, IL-2R*α*, which was also shuttled by both 2D-MSC-MVs and 3D-MSC-MVs. In line with our results, it was recently reported that MSC-MVs showed the presence of insulin like growth factor 1 receptor (IGF-1R) mRNA instead of the ligand IGF-1 mRNA [70]. After being cocultured with IGF-1R containing MSC-MVs, an IGF-1R null fibroblast cell line possessed the ability to express the IGF-1R protein, implying that the IGF-1R mRNA was shuttled from MSC-MVs to target cells and translated into its corresponding protein [70]. Therefore, besides cytokines, more attentions should also be paid to cytokine receptors for better understanding of the possible mechanisms of MSCs.

The potential limitation of our results is that, instead of using autologous integration of MSCs and photoreceptor neurons, we use heterologous integration of MSCs and photoreceptor neurons. However, as immune privileged cells, MSCs were recognized as xenograft, which might reduce the heterogeneous impact. It should be noted that the list of 51 signal factors analyzed in our study was not exhaustive. Attention should be given to additional chemokines, growth factors, and cytokines receptors involved in the regenerative therapies, such as brain-derived neurotrophic factor (BDNF) [32] in future studies. However, our study represents the first reports that assess signal factor secretion in a relatively large scale in different culture conditions.

## 5. Conclusions

Our results revealed that spheroid culture models can improve the ability of MSCs to secret signal factors responsible for anti-inflammation, cell differentiation, and cell survival. In addition, MVs derived from MSCs can stimulate signal factor secretion and 3D-MSC-MVs were superior to 2D-MSC-MVs in stimulating signal factor secretion.

## Supplementary Material

Table S1: Signal factors analyzed via the bead-based assay.
Figure S1: The expression of VEGF and TGFβ3 in supernatant of 2D-MSCs/3D-MSCs and 661W-Sph-25k-MVs co-culture system.

## Figures and Tables

**Table 1:** 15 signal factors secreted by 2D-MSCs.

Symbol	Concentration (pg/mL)
IL-8	6301.9 ± 248.0
IL-6	3595.6 ± 384.9
GRO*α*	2178.5 ± 244.8
TGF*β*1	425.6 ± 27.8
bFGF	408.3 ± 41.6
SCGF-*β*	275.1 ± 23.0
HGF	91.0 ± 15.3
MCP-1	64.1 ± 9.1
SDF-1*α*	45.9 ± 6.8
TGF*β*2	34.6 ± 5.1
G-CSF	19.6 ± 3.6
IL-1*α*	12.5 ± 2.0
IL-1*β*	17.6 ± 1.9
LIF	1.1 ± 0.3
IL-2R*α*	1.0 ± 0.3

Data presented as mean ± standard deviation.

**Table 2:** 21 signal factors secreted by 3D-MSCs.

Symbol	Form							
	Sph-2.5k DC	Sph-2.5k	Sph-6.5k DC	Sph-6.5k	Sph-25k DC	Sph-25k	Sph-50k DC	Sph-50k
	pg/mL							
*IL-1Ra*	0.9 ± 0.1	1.3 ± 0.2	3.0 ± 0.6^*∗*^	3.6 ± 0.4^*∗*^	4.0 ± 0.8^*∗*^	4.4 ± 0.4^*∗*^	4.0 ± 0.6^*∗*^	4.9 ± 0.6^*∗*^
*IL-7*	57.6 ± 6.0^*∗*^	56.3 ± 6.9^*∗*^	107.5 ± 8.6^*∗*^	126.4 ± 10.5^*∗*^	101.3 ± 9.0^*∗*^	106.2 ± 12.9^*∗*^	106.2 ± 11.0^*∗*^	76.1 ± 7.7^*∗*^
*IL-16*	2.2 ± 0.4	4.4 ± 1.3	31.5 ± 6.9^*∗*^	45.3 ± 7.6^*∗*^	6.3 ± 1.2	7.9 ± 1.4	13.3 ± 2.5	13.3 ± 2.3
*MCP-3*	9.4 ± 1.2	12.9 ± 2.0^*∗*^	13.8 ± 2.3^*∗*^	31.8 ± 7.6^*∗*^	10.3 ± 2.0	12.1 ± 3.4^*∗*^	15.6 ± 3.0^*∗*^	18.3 ± 1.8^*∗*^
*VEGF*	4.2 ± 1.5	7.3 ± 1.4	10.3 ± 2.0	10.3 ± 1.5	12.6 ± 2.7^*∗*^	17.3 ± 2.2^*∗*^	23.5 ± 3.3^*∗*^	30.7 ± 7.6^*∗*^
*TGFβ3*	9.3 ± 1.5	25.6 ± 6.1^*∗*^	15.8 ± 1.8	37.2 ± 5.1^*∗*^	35.8 ± 5.3^*∗*^	34.2 ± 7.1^*∗*^	35.2 ± 4.8^*∗*^	38.8 ± 5.4^*∗*^
IL-2R*α*	8.7 ± 1.9^*∗*^	9.4 ± 1.3^*∗*^	13.3 ± 2.1^*∗*^	14.8 ± 2.2^*∗*^	8.2 ± 1.6^*∗*^	10.3 ± 2.9^*∗*^	9.0 ± 1.4^*∗*^	9.3 ± 1.6^*∗*^
IL-6	5101.0 ± 228.7^*∗*^	5205.5 ± 260.9^*∗*^	5659.4 ± 291.6^*∗*^	5960.3 ± 204.3^*∗*^	5474.62 ± 266.6^*∗*^	5497.0 ± 234.5^*∗*^	5332.4 ± 257.7^*∗*^	5349.0 ± 313.2^*∗*^
MCP-1	150.3 ± 17.8^*∗*^	150.4 ± 15.0^*∗*^	150.8 ± 20.6^*∗*^	150.1 ± 21.9^*∗*^	162.3 ± 30.0^*∗*^	203.6 ± 35.6^*∗*^	150.5 ± 18.3^*∗*^	156.6 ± 24.1^*∗*^
LIF	15.0 ± 1.3^*∗*^	15.2 ± 1.0^*∗*^	14.7 ± 0.7^*∗*^	15.2 ± 2.2^*∗*^	15.7 ± 1.6^*∗*^	22.0 ± 5.0^*∗*^	14.6 ± 2.4^*∗*^	20.9 ± 6.1^*∗*^
G-CSF	240.1 ± 19.4^*∗*^	245.6 ± 18.4^*∗*^	253.2 ± 18.9^*∗*^	323.2 ± 75.0^*∗*^	263.5 ± 19.9^*∗*^	1053.4 ± 162.3^*∗*^	261.4 ± 26.3^*∗*^	346.2 ± 27.2^*∗*^
SDF-1*α*	86.8 ± 6.2^*∗*^	89.5 ± 7.5^*∗*^	89.5 ± 8.7^*∗*^	164.5 ± 16.2^*∗*^	88.6 ± 7.6^*∗*^	86.6 ± 8.4^*∗*^	117.8 ± 18.4^*∗*^	119.3 ± 11.8^*∗*^
TGF*β*1	285.9 ± 25.1^*∗*^	195.1 ± 13.6^*∗*^	199.3 ± 13.4^*∗*^	187.0 ± 13.9^*∗*^	143.0 ± 21.3^*∗*^	125.3 ± 12.9^*∗*^	109.7 ± 12.7^*∗*^	99.4 ± 11.5^*∗*^
TGF*β*2	11.1 ± 3.5^*∗*^	10.7 ± 2.8^*∗*^	16.7 ± 1.5^*∗*^	12.4 ± 2.9^*∗*^	17.2 ± 1.3^*∗*^	16.4 ± 1.7^*∗*^	17.2 ± 2.3^*∗*^	12.6 ± 3.2^*∗*^
IL-1*α*	11.3 ± 3.4	19.9 ± 3.1	7.3 ± 2.0	7.5 ± 1.4	10.2 ± 2.3	12.5 ± 2.9	7.2 ± 2.1	8.7 ± 2.5
IL-1*β*	7.3 ± 1.6^*∗*^	8.2 ± 1.8	8.2 ± 2.1	6.3 ± 2.0^*∗*^	14.2 ± 2.8	18.4 ± 2.2	22.1 ± 4.3	22.4 ± 4.0
IL-8	2155.5 ± 70.8^*∗*^	1006.9 ± 242.4^*∗*^	4741.2 ± 107.0^*∗*^	4791.4 ± 232.7^*∗*^	4741.2 ± 166.9^*∗*^	4059.8 ± 162.5^*∗*^	6795.4 ± 314.2	7495.1 ± 337.2
GRO*α*	1804.3 ± 212.2	1862.0 ± 211.3	2503.2 ± 160.0	2648.0 ± 226.3	1985.0 ± 287.4	1751.6 ± 197.7	2045.0 ± 227.5	2435.6 ± 249.0
HGF	65.2 ± 5.9	78.3 ± 10.1	65.0 ± 10.4	45.5 ± 7.1^*∗*^	94.0 ± 10.2	92.2 ± 8.8	86.2 ± 8.2	73.0 ± 5.7
bFGF	275.1 ± 17.6^*∗*^	344.1 ± 24.4	293.5 ± 29.3^*∗*^	164.7 ± 23.0^*∗*^	416.3 ± 16.1	200.6 ± 27.9^*∗*^	328.6 ± 11.4	196.4 ± 16.5^*∗*^
SCGF-*β*	223.4 ± 19.9	230.8 ± 17.9	240.3 ± 29.0	255.4 ± 26.2	284.3 ± 19.1	221.2 ± 22.3	279.4 ± 25.5	268.5 ± 26.9

Data presented as mean ± standard deviation.

^*∗*^ *p* < 0.05 between 2D-MSCs and certain 3D-MSCs groups.

## References

[1] Kim Y., Lee S. H., Kang B., Kim W. H., Yun H., Kweon O. (2016). Comparison of osteogenesis between adipose-derived mesenchymal stem cells and their sheets on poly-*ε*-caprolactone/*β*-tricalcium phosphate composite scaffolds in canine bone defects. *Stem Cells International*.

[2] Cao J., Hou S., Ding H. (2016). In vivo tracking of systemically administered allogeneic bone marrow mesenchymal stem cells in normal rats through biolunimescence imaging. *Stem Cells International*.

[3] An J. H., Park H., Song J. A. (2013). Transplantation of human umbilical cord blood-derived mesenchymal stem cells or their conditioned medium prevents bone loss in ovariectomized nude mice. *Tissue Engineering—Part A*.

[4] Lindenmair A., Hatlapatka T., Kollwig G. (2012). Mesenchymal stem or stromal cells from amnion and umbilical cord tissue and their potential for clinical applications. *Cells*.

[5] Pan G.-H., Chen Z., Xu L. (2016). Low-dose tacrolimus combined with donor-derived mesenchymal stem cells after renal transplantation: a prospective, non-randomized study. *Oncotarget*.

[6] Ko J. H., Lee H. J., Jeong H. J. (2016). Mesenchymal stem/stromal cells precondition lung monocytes/macrophages to produce tolerance against allo- and autoimmunity in the eye. *Proceedings of the National Academy of Sciences of the United States of America*.

[7] Chen Y., Tang Y., Long W., Zhang C. (2016). Stem cell-released microvesicles and exosomes as novel biomarkers and treatments of diseases. *Stem Cells International*.

[8] Jinfeng L., Yunliang W., Xinshan L. (2016). The effect of MSCs derived from the human umbilical cord transduced by fibroblast growth factor-20 on Parkinson’s disease. *Stem Cells International*.

[9] Guiducci S., Manetti M., Romano E. (2011). Bone marrow-derived mesenchymal stem cells from early diffuse systemic sclerosis exhibit a paracrine machinery and stimulate angiogenesis in vitro. *Annals of the Rheumatic Diseases*.

[10] Becker S., Jayaram H., Limb G. A. (2012). Recent advances towards the clinical application of stem cells for retinal regeneration. *Cells*.

[11] Gramlich O. W., Burand A. J., Brown A. J., Deutsch R. J., Kuehn M. H., Ankrum J. A. (2016). Cryopreserved mesenchymal stromal cells maintain potency in a retinal ischemia/reperfusion injury model: toward an off-the-shelf therapy. *Scientific Reports*.

[12] Tsuruma K., Yamauchi M., Sugitani S. (2014). Progranulin, a major secreted protein of mouse adipose-derived stem cells, inhibits light-induced retinal degeneration. *Stem Cells Translational Medicine*.

[13] Tzameret A., Sher I., Belkin M. (2015). Epiretinal transplantation of human bone marrow mesenchymal stem cells rescues retinal and vision function in a rat model of retinal degeneration. *Stem Cell Research*.

[14] Johnson T. V., Dekorver N. W., Levasseur V. A. (2014). Identification of retinal ganglion cell neuroprotection conferred by platelet-derived growth factor through analysis of the mesenchymal stem cell secretome. *Brain*.

[15] Tsai K. S., Yang S. H., Lei Y. P. (2011). Mesenchymal stem cells promote formation of colorectal tumors in mice. *Gastroenterology*.

[16] Bartosh T. J., Ylöstalo J. H., Mohammadipoor A. (2010). Aggregation of human mesenchymal stromal cells (MSCs) into 3D spheroids enhances their antiinflammatory properties. *Proceedings of the National Academy of Sciences of the United States of America*.

[17] Yeh H.-Y., Liu B.-H., Hsu S.-H. (2012). The calcium-dependent regulation of spheroid formation and cardiomyogenic differentiation for MSCs on chitosan membranes. *Biomaterials*.

[18] Pant S., Hilton H., Burczynski M. E. (2012). The multifaceted exosome: biogenesis, role in normal and aberrant cellular function, and frontiers for pharmacological and biomarker opportunities. *Biochemical Pharmacology*.

[19] Cocucci E., Meldolesi J. (2015). Ectosomes and exosomes: shedding the confusion between extracellular vesicles. *Trends in Cell Biology*.

[20] Tseng T.-C., Hsu S.-H. (2014). Substrate-mediated nanoparticle/gene delivery to MSC spheroids and their applications in peripheral nerve regeneration. *Biomaterials*.

[21] Cocucci E., Racchetti G., Meldolesi J. (2009). Shedding microvesicles: artefacts no more. *Trends in Cell Biology*.

[22] Lee Y., El Andaloussi S., Wood M. J. A. (2012). Exosomes and microvesicles: extracellular vesicles for genetic information transfer and gene therapy. *Human Molecular Genetics*.

[23] Bobis-Wozowicz S., Kmiotek K., Sekula M. (2015). Human induced pluripotent stem cell-derived microvesicles transmit RNAs and proteins to recipient mature heart cells modulating cell fate and behavior. *STEM CELLS*.

[24] Deng W., Wang Y., Liu Z., Cheng H., Xue Y. (2014). HemI: a toolkit for illustrating heatmaps. *PLOS ONE*.

[25] Felger J. C., Lotrich F. E. (2013). Inflammatory cytokines in depression: neurobiological mechanisms and therapeutic implications. *Neuroscience*.

[26] Helmy A., Carpenter K. L. H., Menon D. K., Pickard J. D., Hutchinson P. J. A. (2011). The cytokine response to human traumatic brain injury: temporal profiles and evidence for cerebral parenchymal production. *Journal of Cerebral Blood Flow and Metabolism*.

[27] Bhang S. H., Cho S.-W., La W.-G. (2011). Angiogenesis in ischemic tissue produced by spheroid grafting of human adipose-derived stromal cells. *Biomaterials*.

[28] Xie L., Mao M., Zhou L., Jiang B. (2016). Spheroid mesenchymal stem cells and mesenchymal stem cell-derived microvesicles: two potential therapeutic strategies. *Stem Cells and Development*.

[29] Huang G.-S., Dai L.-G., Yen B. L., Hsu S.-H. (2011). Spheroid formation of mesenchymal stem cells on chitosan and chitosan-hyaluronan membranes. *Biomaterials*.

[30] Shi C., Pamer E. G. (2011). Monocyte recruitment during infection and inflammation. *Nature Reviews Immunology*.

[31] Tassoni A., Gutteridge A., Barber A. C., Osborne A., Martin K. R. (2015). Molecular mechanisms mediating retinal reactive gliosis following bone marrow mesenchymal stem cell transplantation. *Stem Cells*.

[32] Emre E., Yüksel N., Duruksu G. (2015). Neuroprotective effects of intravitreally transplanted adipose tissue and bone marrow-derived mesenchymal stem cells in an experimental ocular hypertension model. *Cytotherapy*.

[33] Liao W., Pham V., Liu L. (2016). Mesenchymal stem cells engineered to express selectin ligands and IL-10 exert enhanced therapeutic efficacy in murine experimental autoimmune encephalomyelitis. *Biomaterials*.

[34] Ezquer M., Urzua C. A., Montecino S., Leal K., Conget P., Ezquer F. (2016). Intravitreal administration of multipotent mesenchymal stromal cells triggers a cytoprotective microenvironment in the retina of diabetic mice. *Stem Cell Research and Therapy*.

[35] Manuguerra-Gagné R., Boulos P. R., Ammar A. (2013). Transplantation of mesenchymal stem cells promotes tissue regeneration in a glaucoma model through laser-induced paracrine factor secretion and progenitor cell recruitment. *Stem Cells*.

[36] Stern J. H., Temple S. (2011). Stem cells for retinal replacement therapy. *Neurotherapeutics*.

[37] Hartman Z. C., Poage G. M., Den Hollander P. (2013). Growth of triple-negative breast cancer cells relies upon coordinate autocrine expression of the proinflammatory cytokines IL-6 and IL-8. *Cancer Research*.

[38] Ning Y., Manegold P. C., Hong Y. K. (2011). Interleukin-8 is associated with proliferation, migration, angiogenesis and chemosensitivity in vitro and in vivo in colon cancer cell line models. *International Journal of Cancer*.

[39] Sarvaiya P. J., Guo D., Ulasov I., Gabikian P., Lesniak M. S. (2013). Chemokines in tumor progression and metastasis. *Oncotarget*.

[40] Ylöstalo J. H., Bartosh T. J., Coble K., Prockop D. J. (2012). Human mesenchymal stem/stromal cells cultured as spheroids are self-activated to produce prostaglandin E2 that directs stimulated macrophages into an anti-inflammatory phenotype. *Stem Cells*.

[41] Wang W., Itaka K., Ohba S. (2009). 3D spheroid culture system on micropatterned substrates for improved differentiation efficiency of multipotent mesenchymal stem cells. *Biomaterials*.

[42] Bartosh T. J., Ylöstalo J. H., Bazhanov N., Kuhlman J., Prockop D. J. (2013). Dynamic compaction of human mesenchymal stem/precursor cells into spheres self-activates caspase-dependent il1 signaling to enhance secretion of modulators of inflammation and immunity (PGE2, TSG6, and STC1). *Stem Cells*.

[43] Akitsu A., Ishigame H., Kakuta S. (2015). IL-1 receptor antagonist-deficient mice develop autoimmune arthritis due to intrinsic activation of IL-17-producing CCR2^+^V*γ*6^+^*γδ*T cells. *Nature Communications*.

[44] Chazen G. D., Pereira G. M. B., LeGros G., Gillis S., Shevach E. M. (1989). Interleukin 7 is a T-cell growth factor. *Proceedings of the National Academy of Sciences of the United States of America*.

[45] Raposo R. A. S., Trudgian D. C., Thomas B., Van Wilgenburg B., Cowley S. A., James W. (2011). Protein kinase C and NF-*κ*B-dependent CD4 downregulation in macrophages induced by T cell-derived soluble factors: consequences for HIV-1 infection. *Journal of Immunology*.

[46] Archin N. M., van den Boom L., Perelygina L., Hilliard J. M., Atherton S. S. (2003). Delayed spread and reduction in virus titer after anterior chamber inoculation of a recombinant of HSV-1 expressing IL-16. *Investigative Ophthalmology and Visual Science*.

[47] Maddaluno M., Di Lauro M., Di Pascale A. (2011). Monocyte chemotactic protein-3 induces human coronary smooth muscle cell proliferation. *Atherosclerosis*.

[48] Clatworthy M. R., Harford S. K., Mathews R. J., Smith K. G. C. (2014). Fc*γ*RIIb inhibits immune complex-induced VEGF-A production and intranodal lymphangiogenesis. *Proceedings of the National Academy of Sciences of the United States of America*.

[49] Guaiquil V. H., Pan Z., Karagianni N., Fukuoka S., Alegre G., Rosenblatt M. I. (2014). VEGF-B selectively regenerates injured peripheral neurons and restores sensory and trophic functions. *Proceedings of the National Academy of Sciences of the United States of America*.

[50] Morille M., Toupet K., Montero-Menei C. N., Jorgensen C., Noël D. (2016). PLGA-based microcarriers induce mesenchymal stem cell chondrogenesis and stimulate cartilage repair in osteoarthritis. *Biomaterials*.

[51] Yoon H. H., Bhang S. H., Shin J.-Y., Shin J., Kim B.-S. (2012). Enhanced cartilage formation via three-dimensional cell engineering of human adipose-derived stem cells. *Tissue Engineering—Part A*.

[52] Xin H., Li Y., Buller B. (2012). Exosome-mediated transfer of miR-133b from multipotent mesenchymal stromal cells to neural cells contributes to neurite outgrowth. *Stem Cells*.

[53] Gatti S., Bruno S., Deregibus M. C. (2011). Microvesicles derived from human adult mesenchymal stem cells protect against ischaemia-reperfusion-induced acute and chronic kidney injury. *Nephrology Dialysis Transplantation*.

[54] Xin H., Li Y., Cui Y., Yang J. J., Zhang Z. G., Chopp M. (2013). Systemic administration of exosomes released from mesenchymal stromal cells promote functional recovery and neurovascular plasticity after stroke in rats. *Journal of Cerebral Blood Flow and Metabolism*.

[55] Ju G.-Q., Cheng J., Zhong L. (2015). Microvesicles derived from human umbilical cord mesenchymal stem cells facilitate tubular epithelial cell dedifferentiation and growth via hepatocyte growth factor induction. *PLoS ONE*.

[56] Hiraoka A., Sugimura A., Seki T. (1997). Cloning, expression, and characterization of a cDNA encoding a novel human growth factor for primitive hematopoietic progenitor cells. *Proceedings of the National Academy of Sciences of the United States of America*.

[57] Gehling U. M., Ergün S., Schumacher U. (2000). In vitro differentiation of endothelial cells from AC133-positive progenitor cells. *Blood*.

[58] Ouma C., Keller C. C., Davenport G. C. (2010). A novel functional variant in the stem cell growth factor promoter protects against severe malarial anemia. *Infection and Immunity*.

[59] Williams R. L., Hilton D. J., Pease S. (1988). Myeloid leukaemia inhibitory factor maintains the developmental potential of embryonic stem cells. *Nature*.

[60] Davis S. M., Collier L. A., Leonardo C. C., Seifert H. A., Ajmo C. T., Pennypacker K. R. (2017). Leukemia inhibitory factor protects neurons from ischemic damage via upregulation of superoxide dismutase 3. *Molecular Neurobiology*.

[61] Kirby E. D., Kuwahara A. A., Messer R. L., Wyss-Coray T. (2015). Adult hippocampal neural stem and progenitor cells regulate the neurogenic niche by secreting VEGF. *Proceedings of the National Academy of Sciences of the United States of America*.

[62] Ratajczak J., Wysoczynski M., Hayek F., Janowska-Wieczorek A., Ratajczak M. Z. (2006). Membrane-derived microvesicles: important and underappreciated mediators of cell-to-cell communication. *Leukemia*.

[63] Phinney D. G., Di Giuseppe M., Njah J. (2015). Mesenchymal stem cells use extracellular vesicles to outsource mitophagy and shuttle microRNAs. *Nature Communications*.

[64] Collino F., Deregibus M. C., Bruno S. (2010). Microvesicles derived from adult human bone marrow and tissue specific mesenchymal stem cells shuttle selected pattern of miRNAs. *PLoS ONE*.

[65] Perez V. L., Caspi R. R. (2015). Immune mechanisms in inflammatory and degenerative eye disease. *Trends in Immunology*.

[66] Kaneko H., Dridi S., Tarallo V. (2011). DICER1 deficit induces *Alu* RNA toxicity in age-related macular degeneration. *Nature*.

[67] Murakami Y., Matsumoto H., Roh M. (2014). Programmed necrosis, not apoptosis, is a key mediator of cell loss and DAMP-mediated inflammation in dsRNA-induced retinal degeneration. *Cell Death and Differentiation*.

[68] Yu B., Shao H., Su C. (2016). Exosomes derived from MSCs ameliorate retinal laser injury partially by inhibition of MCP-1. *Scientific Reports*.

[69] Hoh B. L., Hosaka K., Downes D. P. (2011). Monocyte chemotactic protein-1 promotes inflammatory vascular repair of murine carotid aneurysms via a macrophage inflammatory protein-1*α* and macrophage inflammatory protein-2-dependent pathway. *Circulation*.

[70] Tomasoni S., Longaretti L., Rota C. (2013). Transfer of growth factor receptor mRNA via exosomes unravels the regenerative effect of mesenchymal stem cells. *Stem Cells and Development*.

