# Supplementary material for: Signal Factors Secreted by 2D and Spheroid Mesenchymal Stem Cells and by Cocultures of Mesenchymal Stem Cells Derived Microvesicles and Retinal Photoreceptor Neurons
Source: Stem Cells Int. 2017 Jan 18;2017:2730472. doi: 10.1155/2017/2730472 (PMC5286488; doi:10.1155/2017/2730472)
Supplement: Supplementary file 1 — Table S1: Signal factors analyzed via the bead-based assay. Figure S1: The expression of VEGF and TGFβ3 in supernatant of 2D-MSCs/3D-MSCs and 661W-Sph-25k-MVs co-culture system. [file 2730472.f1.doc]

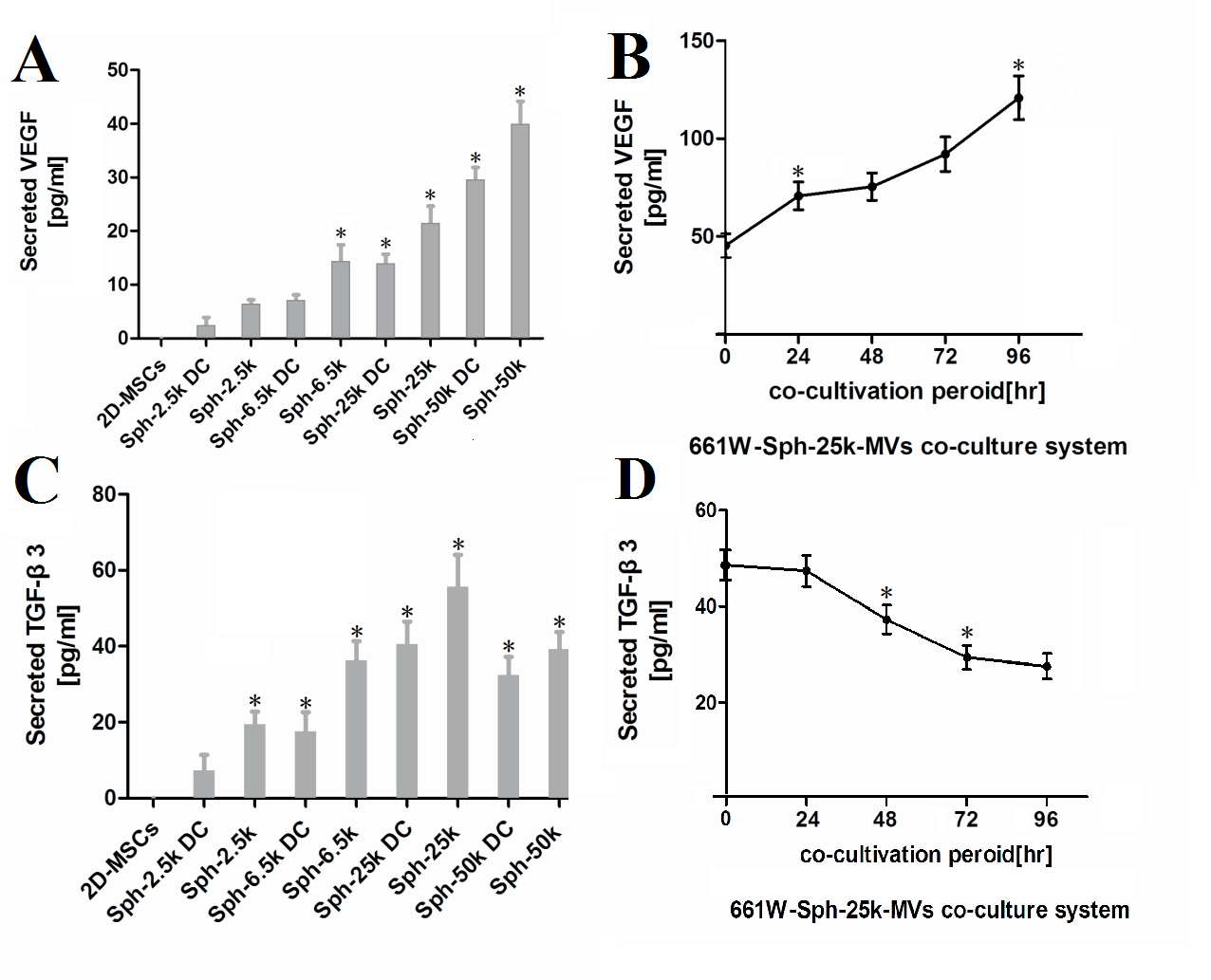


Supplementary Figure S1. The expression of VEGF and TGFβ3 in supernatant of 2D-MSCs/3D-MSCs and 661W-Sph-25k-MVs co-culture system were detected. (A-D) The expression of VEGF and TGFβ3 in supernatant of 2D-MSCs/3D-MSCs (A, C) and 661W-Sph-25k-MVs co-culture system (B, D) were quantified by ELISA. * denote significant differences of p value less than 0.05.

**Table S1. Signal factors analyzed via the bead-based assay**

| **Protein** | **Symbol** | **Function** |
| --- | --- | --- |
| Interleukin 1, alpha | IL-1α | Pro-inflammatory and anti-inflammatory factor  Pro-angiogenic |
| Interleukin 1, beta | IL-1β | Pro-inflammatory factor |
| Interleukin 1 receptor antagonist | IL-1Ra | Inhibits IL1α and IL-1β |
| Interleukin 2 | IL-2 | Regulates proliferation of T and B cells |
| Interleukin 2 receptor, alpha | IL-2Rα | An integral part of interleukin 2 receptor |
| Interleukin 3 | IL-3 | Potent growth promoting cytokine |
| Interleukin 4 | IL-4 | Improves repair  Inhibits IFN-γ |
| Interleukin 5 | IL-5 | Regulates growth and differentiation of B cells and eosinophils |
| Interleukin 6 | IL-6 | Pro-inflammatory and anti-inflammatory factor |
| Interleukin 7 | IL-7 | Regulates B and T cell development  Pro-angiogenic |
| Interleukin 8 | IL-8 | Chemoattractant  Potent angiogenic factor |
| Interleukin 9 | IL-9 | Stimulates cell proliferation prevents apoptosis |
| Interleukin 10 | IL-10 | Anti-inflammatory factor |
| Interleukin 12 p70 | IL-12 p70 | Pro-inflammatory factor |
| Interleukin 12 p40 | IL-12 p40 | Pro-inflammatory factor |
| Interleukin 13 | IL-13 | Anti-inflammatory factor |
| Interleukin 15 | IL-15 | Regulates T and NK cell activation and proliferation |
| Interleukin 16 | IL-16 | Chemoattractant  Regulates T cell activation |
| Interleukin 17 | IL-17 | Pro-inflammatory factor |
| Interleukin 18 | IL-18 | Pro-inflammatory factor |
| Monocyte chemoattractant protein 1 | MCP-1 | Chemotactic for monocytes and basophils |
| Monocyte chemoattractant protein 3 | MCP-3 | Chemotactic for macrophages |
| Growth-regulated oncogene α | GROα | Chemotactic for neutrophils  Pro-growth of certain tumor |
| Cutaneous T cell-attracting chemokine | CTACK | Chemotactic for T cells mediates skin inflammation |
| Regulated upon activation normal T cell expressed and secreted factor | RANTES | Chemotactic for T helper cells and eosinophils |
| Eotaxin | CCL11 | Pro-inflammatory factor |
| Monokine induced by interferon-γ | MIG | Chemotactic for lymphocytes but not for neutrophils |
| Macrophage inflammatory protein 1 alpha | MIP-1α | Pro-inflammatory factor  Chemoattractant |
| Macrophage inflammatory protein 1 beta | MIP-1β | Pro-inflammatory factor  Chemoattractant |
| Macrophage migration inhibition factor | MIF | Regulates function of macrophage |
| Leukemia inhibitory factor | LIF | Regulates hematopoietic and neuronal cell differentiation and immune tolerance |
| Macrophage colony-stimulating factor | M-CSF | Controls production, differentiation and function of macrophages |
| Granulocyte colony stimulating factor | G-CSF | Controls production, differentiation and function of granulocytes |
| Granulocyte-macrophage colony stimulating factor | GM-CSF | Regulates the production, differentiation and function of granulocytes and macrophages |
| Interferon-inducible protein-10 | IP-10 | Stimulates monocytes, NK cells and T cells |
| Interferon, alpha 2 | IFN-α2 | Responds to viral infection |
| Interferon-gamma | IFN-γ | Pro-inflammatory factor  Responds to viral infection |
| Tumor necrosis factor alpha | TNF-α | Pro-inflammatory factor |
| Tumor necrosis factor b | TNF-b | Pro-inflammatory factor |
| Tumor necrosis factor related apoptosis inducing ligand | TRAIL | Induces apoptosis |
| Transforming growth factor, beta 1 | TGFβ1 | Regulates proliferation, differentiation and adhesion |
| Transforming growth factor, beta 2 | TGFβ2 | Regulates proliferation, differentiation and adhesion |
| Transforming growth factor, beta 3 | TGFβ3 | Regulates embryogenesis and cell differentiation |
| Stem cell factor | SCF | Regulates cell migration |
| Hepatocyte growth factor | HGF | Regulates cell growth, cell motility and morphogenesis |
| Fibroblast growth factor 2 (basic) | bFGF | Mitogenic and angiogenic factor |
| Stromal cell derived factor-1α | SDF-1α | Regulates embryogenesis, immune surveillance and inflammation response |
| Beta nerve growth factor | b-NGF | Stimulates nerve growth |
| Platelet-derived growth factor BB | PDGF-BB | Mitogenic factor |
| Vascular endothelial growth factor | VEGF | Inducing angiogenesis, vasculogenesis and endothelial cell growth |
| Stem cell growth factor, beta | SCGF-β | Growth factor for hematopoietic progenitor cells |
